# A rare case of isolated nasal tip neurofibroma in an 11-year-old female without neurofibromatosis: A case report

**DOI:** 10.1016/j.ijscr.2024.110712

**Published:** 2024-12-01

**Authors:** Abdullah Fadhel Almusallam, Mosab Tareq Atmeh, Mohammed Bader Obeidat, Hasan Zuhair El-Isa, Ahmed Smadi, Mousa Tarek Atmeh

**Affiliations:** a Kuwait Ministry of Health, Kuwait; b Jordan University of Science and Technology, Jordan; c Department of Oncology, Royal Medical Services, Jordan; d Internal Medicine Department, Royal Medical Services, Jordan; e Department of Otolaryngology, Royal Medical Services, Jordan

**Keywords:** Neurofibroma, Nasal tip mass, Nasal lesion excision, Neurofibromatosis

## Abstract

**Introduction and importance:**

Isolated neurofibromas of the nasal tip are uncommon, particularly in pediatric patients. Neurofibromas are benign tumors that arise from the peripheral nerve sheath and are usually associated with neurofibromatosis type 1 (NF1). Isolated cases present unique challenges due to their location and the importance of cosmetic outcomes. This case highlights the presentation, diagnostic process, and management of a rare nasal tip neurofibroma in an 11-year-old girl.

**Case presentation:**

An 11-year-old female presented with a gradually enlarging mass on the nasal tip over several months, causing cosmetic concern without pain, bleeding, or obstruction. Clinical examination revealed a firm, non-tender lesion, about 1 cm in diameter, with normal skin. Imaging confirmed a well-defined mass localized to the nasal tip. Surgical excision was performed, and histopathology confirmed a diagnosis of neurofibroma. Follow-up showed no recurrence, and the patient was satisfied with the cosmetic result.

**Clinical discussion:**

Neurofibromas, though benign, can cause aesthetic concerns, particularly in prominent areas like the nasal tip. Isolated neurofibromas in children without NF1 are rare. Surgical excision is the treatment of choice, with emphasis on complete removal to prevent recurrence. This case demonstrates successful excision with clear margins, preserving nasal structure and appearance. Long-term monitoring is essential for recurrence prevention.

**Conclusion:**

Isolated neurofibroma of the nasal tip is a rare condition in children. Surgical excision remains the treatment of choice, with careful planning required to preserve both cosmetic and functional outcomes. Regular follow-up is crucial to monitor for recurrence, especially in the absence of neurofibromatosis.

## Highlights

• Neurofibromas occurring in the nasal tip are rare, especially in pediatric patients.
• Histopathological examination and immunohistochemical analysis confirmed the diagnosis of neurofibroma.
• Managing nasal neurofibromas is challenging, balancing tumor excision with preserving the nasal tip's appearance.
• Ensuring complete removal of the neurofibroma is crucial to prevent recurrence.
• Due to the rarity of this condition and the potential for recurrence, long-term follow-up is recommended.

## 1 Background

Neurofibromas are benign tumors originating from the peripheral nerve sheath, comprising Schwann cells, fibroblasts, perineural cells, and axons [1]. These tumors may occur sporadically or in association with neurofibromatosis type 1 (NF1), an autosomal dominant genetic disorder. While neurofibromas typically occur in the context of NF1, isolated neurofibromas without signs of NF1 are rare, particularly in pediatric patients [2]. Isolated neurofibromas of the nasal tip present unique challenges due to their location, potential for recurrence, and the need for cosmetic considerations.

## 2 Introduction

Neurofibromas can present in a localized or diffuse form. The localized form, as seen in this case, is more common and involves a defined area, whereas the diffuse form involves extensive skin and subcutaneous tissue. Isolated nasal neurofibromas are exceedingly rare, especially in children, and pose challenges in diagnosis and management due to the cosmetic and functional significance of the nasal tip [3]. This case report discusses an 11-year-old girl with an isolated neurofibroma of the nasal tip, presenting without other signs of NF1 [4].

## 3 Case report

An 11-year-old female with no personal or family history of neurofibromatosis presented to the ENT clinic with a gradually enlarging mass on her nasal tip, which had been noticeable for several months. The patient and her family were primarily concerned with the cosmetic appearance of the mass, as it had become more prominent over time. There were no associated symptoms such as pain, bleeding, or nasal obstruction. The family was worried about the nature of the lesion, fearing it might be malignant. Upon clinical examination, a firm, non-tender, well-circumscribed, mobile lesion approximately 1 cm in diameter was identified at the nasal tip, with normal overlying skin. No signs of ulceration, discoloration, or discharge were noted. The patient exhibited no other symptoms, such as nasal congestion or respiratory issues, and further examination revealed no additional cutaneous lesions suggestive of neurofibromatosis, such as café-au-lait spots. A comprehensive head and neck examination showed no abnormalities, and the patient did not display any neurological or ocular deficits.

## 4 Diagnostic assessment

**Fig. 1:**
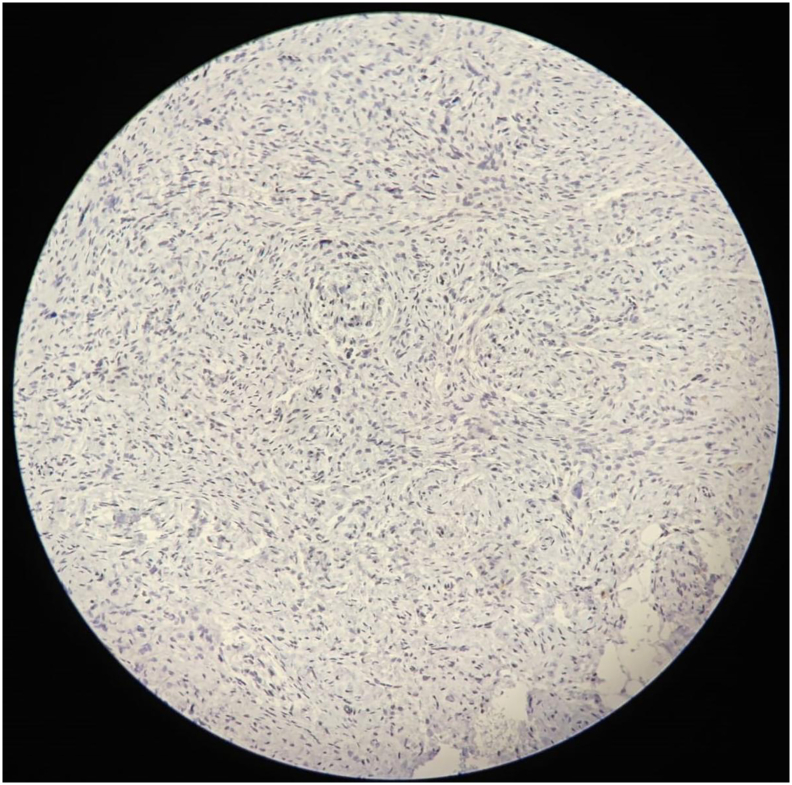
Immunohistochemical staining with cytokeratin (CK) showing negative epithelial differentiation, excluding an epithelial tumor.
• **Smooth Muscle Actin (SMA):** Negative, ruling out a smooth muscle tumor.
• **CD34 (**Fig. 2**):** Negative, excluding a vascular tumor.

Histopathological examination of the excised tissue revealed a spindle cell neoplasm within a myxoid stroma. The spindle cells had elongated, wavy nuclei, characteristic of a neural origin. Immunohistochemical analysis revealed:

• **S100:** Patchy positivity, consistent with a neural origin.
• **Cytokeratin (CK) (**Fig. 1**):** Negative, excluding epithelial differentiation.

**Fig. 2:**
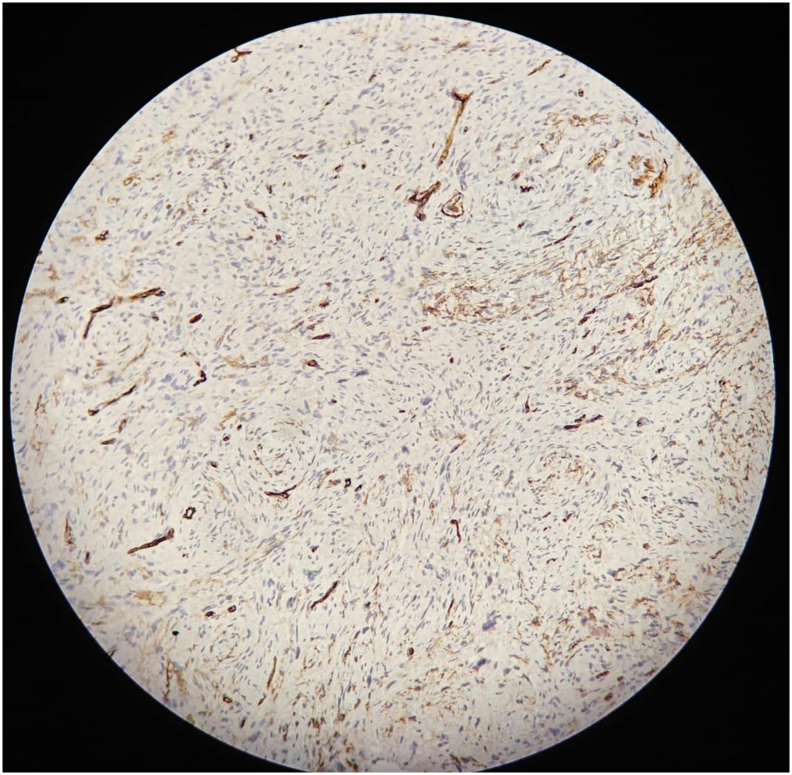
Immunohistochemical staining with CD34 showing negative results, ruling out a vascular tumor.

These findings were consistent with a diagnosis of neurofibroma, with no evidence of malignancy or atypical cells present.

## 5 Timeline

• **September 2023:** The patient first noticed a small mass on her nasal tip.
• **December 2023:** The mass became more noticeable, leading to a visit to the ENT clinic.
• **January 2024:** A firm, non-tender mass on the nasal tip was identified during clinical examination, and the patient was referred for imaging.
• **Mid-January 2024:** Imaging studies (CT and MRI) showed a well-defined mass confined to the nasal tip.
• **Late January 2024:** Surgical excision of the mass was performed. Histopathology confirmed neurofibroma.
• **February 2024:** Postoperative follow-up showed no signs of recurrence, and the patient was satisfied with the cosmetic outcome.
• **April 2024:** A second follow-up showed no recurrence, and the patient remained asymptomatic with no new lesions.

## 6 Discussion

Nasal tip lesions, particularly those affecting facial aesthetics, are often brought to medical attention early. In this case, the cosmetic appearance of the nasal mass prompted the patient's family to seek care [5]. Isolated neurofibromas of the nasal tip are rare, and diagnosis often requires imaging and histopathological analysis, as mentioned above in the diagnostic assessment section, to confirm the benign nature of the lesion [6]. Complete surgical excision with clear margins is critical to prevent recurrence [7].

The lesion in this patient was localized to the subcutaneous region and did not involve the lower lateral cartilage, allowing for a straightforward excision without compromising the lower lateral cartilage. Preoperative imaging confirmed that the lesion's boundaries did not reach the lower structures such as lower lateral cartilage. In cases where larger lesions involve the lower lateral cartilage, partial excision of the affected cartilage may be necessary. Reconstruction using conchal cartilage from the auricle or costal cartilage from the ribs can be used to restore the nasal form and function while preserving the aesthetic outcome. Given the prominence of the nasal tip in facial aesthetics, particular care was taken to preserve symmetry and avoid deformity, which is especially important in pediatric patients with ongoing nasal development. Postoperative follow-up showed that the lesion was successfully excised with no recurrence noted at follow-up.

The management of nasal neurofibromas presents challenges, particularly in achieving a balance between complete tumor excision and cosmetic preservation of the nasal tip, which plays a significant role in facial aesthetics [9]. Preoperative imaging is essential in evaluating the extent of the tumor, and for small, well-localized lesions like this case, a straightforward excision approach is often sufficient [10].

## 7 Conclusion

Isolated neurofibromas of the nasal tip, although rare, require a tailored surgical approach to ensure complete removal while preserving cosmetic outcomes. In this case, surgical excision achieved clear margins and a favorable cosmetic result, with no recurrence at follow-up. The patient as well as her parents was satisfied with the cosmetic outcome. Due to the rarity of this condition and the potential for recurrence, long-term follow-up is advised. Preoperative assessment and careful surgical planning are crucial to achieving optimal outcomes in such cases.

## Methods

This case report has been written in accordance with the SCARE 2023 guidelines [11].

## Informed consent

Written informed consent was obtained from the patient's parents for the publication of this case report. The patient's parents were informed that no identifying information would be disclosed.

## Ethical approval

According to the policies of the institute where the case report was conducted, ethical approval is not required for case reports, and therefore, they are exempt from ethical approval.

## Guarantor

Abdullah Fadhel Almusallam

## Research registration number

Not applicable here.

## Funding

No funding was received for the preparation of this case report.

## Author contribution

Abdullah Fadhel Almusallam: Literature review, writing, editing and manuscript drafting.

Mosab Tareq Atmeh: Literature review, writing, editing and manuscript drafting.

Mohammed Bader Obeidat: Patient management planning, contribution to the diagnostic process and management of the case, as well as general mentorship.

Hasan Zuhair El-Isa: Literature review, writing and editing.

Ahmed Smadi: Patient management planning, critical review, supervision, and final approval.

Mousa T. Atmeh: Patient management planning and critical review.

## Conflict of interest statement

The authors declare no conflicts of interest regarding the publication of this case report.
